# Enteric pathogens relationship with small bowel histologic features of environmental enteric dysfunction in a multicountry cohort study

**DOI:** 10.1016/j.ajcnut.2024.02.026

**Published:** 2024-09-17

**Authors:** Najeeha T Iqbal, Sarah Lawrence, Tahmeed Ahmed, Kanta Chandwe, Shah M Fahim, Eric R Houpt, Furqan Kabir, Paul Kelly, Jie Liu, Mustafa Mahfuz, Monica Mweetwa, Kelley VanBuskirk, Phillip I Tarr, Donna M Denno, EEDBI Consortium, Kumail Ahmed, Sheraz Ahmed, Md. Ashraful Alam, S. Asad Ali, Beatrice Amadi, Subhasish Das, Md. Amran Gazi, Rashidul Haque, Md. Mehedi Hasan, Md. Shabab Hossain, Aneeta Hotwani, Shahneel Hussain, Junaid Iqbal, Sadaf Jakhro, Ta-Chiang Liu, Ramendra Nath Mazumder, Christopher A Moskaluk, Abdul Khalique Qureshi, Shyam S Raghavan, Masudur Rahman, Najeeb Rahman, Kamran Sadiq, Shafiqul Alam Sarker, Peter B Sullivan, Guillermo J Tearney, Fayaz Umrani, Omer H Yilmaz, Kanekwa Zyambo

**Affiliations:** 11 Department of Paediatrics and Child Health, Aga Khan University, Karachi, Pakistan; 12 International Centre for Diarrhoeal Disease Research, Bangladesh, Dhaka, Bangladesh; 13 Department of Biological and Biomedical Sciences, Aga Khan University, Karachi, Pakistan; 14 Tropical Gastroenterology and Nutrition Group, University of Zambia School of Medicine, Lusaka, Zambia; 15 Infectious Diseases Division, International Centre for Diarrhoeal Disease Research, Bangladesh, Dhaka, Bangladesh; 16 Department of Pathology and Immunology, Washington University, St. Louis, MO, USA; 17 Department of Pathology, University of Virginia School of Medicine, Charlottesville, VA, USA; 18 Department of Gastroenterology, Sheikh Russel National Gastroliver Institute and Hospital, Dhaka, Bangladesh; 19 Department of Paediatrics, Children's Hospital, University of Oxford, Oxford, UK; 20 Department of Pathology, Harvard Medical School, Boston, MA, USA; 21 Department of Pathology, Massachusetts General Hospital, Boston, MA, USA; 22 Tropical Gastroenterology & Nutrition group, University of Zambia School of Medicine, Lusaka, Zambia; 1 Department of Paediatrics and Child Health, Aga Khan University, Karachi, Pakistan; 2 Department of Pediatrics, University of Washington, Seattle, WA, United States; 3 Nutrition Research Division, International Centre for Diarrhoeal Disease Research, Bangladesh, Dhaka, Bangladesh; 4 Tropical Gastroenterology and Nutrition Group, University of Zambia, Lusaka, Zambia; 5 Division of Infectious Diseases and International Health, University of Virginia, Charlottesville, VA, United States; 6 Blizard Institute, Barts and The London School of Medicine and Dentistry, Queen Mary University of London, London, United Kingdom; 7 School of Public Health, Qingdao University, Qingdao, China; 8 Department of Global Health, University of Washington, Seattle, WA, United States; 9 Department of Pediatrics, Washington University, St. Louis, MO, United States

**Keywords:** enteropathogens, qPCR, TAC, environmental enteric dysfunction, virulence loci, LMIC

## Abstract

**Background:**

Environmental Enteric Dysfunction (EED) is an acquired disorder of asymptomatic altered gut function, the etiology of which is unknown. EED is postulated to be a major contributor to growth faltering in early childhood in regions where early-life enteropathogenic carriage is prevalent. Few studies have examined the critical organ (the upper small bowel) with enteropathogens in the evolution of small bowel disease.

**Objectives:**

The objective of this study was to determine if fecal enteropathogenic detection predicts subsequent EED histology.

**Methods:**

Fecal samples were obtained from undernourished children aged <2 y without diarrhea enrolled in 3 cohort studies, who failed nutritional intervention and subsequently underwent endoscopy. Duodenal biopsies from 245 (Bangladesh *n* = 120, Pakistan *n* = 57, and Zambia *n* = 68) children were scored using a semiquantitative histologic grading protocol. Thirteen enteropathogens were sought in common across the 3 centers using TaqMan array cards (TAC) (Bangladesh and Pakistan) and the Luminex platform (Zambia). An additional 18 pathogens and 32 virulence loci were sought by TAC and included in sensitivity analyses restricted to TAC data.

**Results:**

Multivariable linear regressions adjusting for study center, age at stool collection, and stool-to-biopsy interval demonstrated the following: *1*) an association of norovirus and *Shigella* detection with subsequent enterocyte injury [β 0.2 (95% CI: 0.1, 0.3); *P* = 0.002 and β 0.2 (95% CI: 0.0, 0.3); *P* = 0.008, respectively], *2*) association of *Campylobacter* with intraepithelial lymphocytes [β 0.2 (95% CI: 0.0, 0.4); *P* = 0.046], and *3*) association of *Campylobacter* and enterotoxigenic *Escherichia coli* with a summative EED histopathology index score [β 4.2 (95% CI: 0.8, 7.7); *P* = 0.017 and β 3.9 (95% CI: 0.5, 7.3); *P* = 0.027, respectively]. All but 2 of these associations (*Shigella*-enterocyte injury and *Campylobacter*-index score) were also demonstrated in TAC-only sensitivity analyses, which identified additional associations between other pathogens, pathogen burden, or virulence loci primarily with the same histologic parameters.

**Conclusions:**

The detection of some enteropathogens in asymptomatic infections is associated with subsequent EED histopathology. These novel findings offer a basis for future EED etiology and pathogenesis studies.

## Introduction

Suboptimal growth in childhood is caused by complex interactions between illness and nutritional, environmental, socioeconomic, and cultural influences [[1], [2], [3]]. One causal factor is environmental enteric dysfunction (EED), a subclinical inflammation of the small intestine manifested by altered villus architecture and increased gut permeability [4]. Our understanding of the pathogenesis of EED has been hampered by the lack of access to pediatric upper-gastrointestinal endoscopy in EED-endemic settings. This series reports pooled analyses of duodenal biopsies from 3 cohorts of undernourished (stunted or wasted) children in Pakistan, Bangladesh, and Zambia, whose undernutrition was refractory to standard intervention and who had no identifiable cause of their growth failure [5]. The biopsies were subjected to a rigorous standardized scoring based on 8 histologic parameters and a global index score comprised of the 5 most discriminatory histologic parameters based on comparison to tissue without any pathologic abnormality [6].

The community of pathogens harbored by the host is collectively termed the pathobiome [7,8]. The influence of early-life microbes profoundly affects the gut ecosystem and the developing immune system [9]. It is not known if 1 or more enteropathogens elicit or influence the EED process. Here, we examined asymptomatic carriage prevalence of enteropathogens across the centers, as well as the relationship of pathogen detection with scores of subsequent histology, to understand if individual pathogens or pathogen burden could predict EED histopathology.

## Methods

### Patient cohorts

Study participants included children enrolled in the studies Biomarkers of Environmental Enteropathy in Children (BEECH), Bangladesh Environmental Enteric Dysfunction (BEED), and Study of Environmental Enteropathy and Malnutrition (SEEM), conducted in Zambia, Bangladesh, and Pakistan, respectively. They underwent esophagogastroduodenoscopy with duodenal biopsy samples collected and had fecal pathogen data available. Detailed study methodologies, including enrollment procedures, eligibility criteria, and recruitment and sampling timeframes, as well as the characteristics of study participants, are described elsewhere [5]. Briefly, the BEECH study included children aged <18 mo who resided in an urban slum in Lusaka, had wasting or stunting insufficiently responsive to nutritional intervention, and underwent esophagogastroduodenoscopy and biopsy. The BEED study enrolled children aged 12–18 mo who resided in an urban slum in Dhaka into 2 groups – “stunted” [length-for-age *z*-score (LAZ) <-2] and “at risk for stunting” (LAZ < -1 to >-2). Children without improvement in their linear growth category after nutritional intervention underwent esophagogastroduodenoscopy and biopsy. In the SEEM study, infants aged 9 mo with wasting from rural Sindh province were provided nutritional intervention; those with insufficient response underwent esophagogastroduodenoscopy and biopsy. Biopsy samples were collected across studies between November 2016 and August 2019.

### Pathogen analyses

#### Stool collection

Stools were collected at field sites, transported in cooler boxes (4ºC) (BEECH and SEEM) or portable liquid nitrogen tanks (BEED) to the research facilities at each study center, and stored at -80°C until further processing. For children with >1 stool, the sample collected closest to the biopsy was analyzed.

#### Fecal analysis using TAC cards

We used customized TaqMan array cards (TAC) to detect pathogens in fecal samples in the BEED (card version B2541-44) and SEEM (card version P3587) studies. Stools were subjected to sequential mechanical disruption by bead-beating and lysis using buffer, followed by the removal of inhibitors, purification, and elution of total nucleic acid (TNA) using the QIAamp Fast DNA Stool Mini kit (Qiagen). Briefly, 180–220 mg stool samples were mixed with InhibitEX buffer containing phocine herpes virus (PhHV; Houpt Laboratory, UVA) and MS2 bacteriophage (MS2; Houpt Laboratory, UVA) extrinsic controls as DNA and RNA targets, respectively, to check the efficiency of extraction, reverse transcription, and amplification. Samples were homogenized for 2 min with ∼370 mg glass beads (Sigma), incubated at 95°C for 5 min, and centrifuged, after which the resulting clear lysate was extracted with 600 μL of InhibitEx and treated with 200 μL of elution buffer for TNA elution.

The TAC protocol was performed as described elsewhere [10]. Briefly, 20 μL of TNA was added to 80 μL of Agpath one-step RT-PCR master mix containing nuclease-free water, 2X Agpath buffer, and enzyme mix (Thermo Fisher Scientific). The card was loaded, centrifuged, and sealed, after which the loading ports were excised and the card was inserted into QuantStudio 7 Flex platform (Thermo Fisher Scientific). The sample was considered a valid positive if 2 conditions were met: the sample’s target cycle threshold (Ct) value was <35, and the reference extraction blank was negative for each target [11]. The extrinsic controls (PhHV and MS2) were required to have Ct values of <35 for a valid negative result. Our TAC cards were customized to detect common bacteria, viruses, and protozoa associated with diarrhea [11,12] and EED [13,14].

#### Fecal analysis using Luminex assay

The BEECH study used the Luminex xTAG gastrointestinal pathogen panel for the simultaneous detection of 13 enteropathogens. It is based on the hybridization of PCR products on the bead with different fluorescence intensities that are directly proportional to the concentration of the amplified product of a particular target gene. Data acquisition and analysis were performed using the MagPix platform. Briefly, samples were extracted using the Qiagen stool DNA extraction kit. The xTAG gastrointestinal pathogen panel assay was performed per the manufacturer's instructions (Luminex Molecular Diagnostics) [15].

Organisms were excluded from analysis if tested at only 1 center. TAC cards included *Mycobacterium tuberculosis* (prevalence of 1.7% across BEED and SEEM), which was excluded from the analysis because it is not considered a cause of the enteric illness of interest. The enteropathogenic targets common to the BEED and SEEM TAC cards are shown in Supplemental Table 1. We also list the 13 pathogens sought in the BEECH study; the targets are proprietary. Supplemental Table 2 attributes individual pathogens based on >1 TAC target. Thirty-one pathogens were available in the BEED/SEEM dataset (including the 13 BEECH pathogens). Pathogen burden was defined as the number of enteropathogens detected in a stool sample. Virulence locus burden was defined for the BEED and SEEM cohorts only, as the number of positive loci or protein targets from a possible 36 (Supplemental Tables 1 and 2).

### Histology scoring of duodenal biopsies

The histology scoring system and procedures are described in a companion paper in this series [6]. Briefly, endoscopic mucosal pinch biopsies from the second or third duodenal segment were sectioned and stained with hematoxylin and eosin. Slide images uploaded to a telepathology platform were semiquantitatively scored across 8 histologic parameters by 2 or 3 Consortium gastrointestinal pathologists (definitions for each parameter and the range of possible scores are displayed in Supplemental Table 3). The slide consensus scores for each parameter were averaged across pathologist readings. Finally, a global index score – the total score percent-5 (TSP-5) – developed to identify EED and determine its histopathologic severity, was calculated from the 5 histologic parameters that were most informative in identifying EED compared with a reference group of American children without a clinical or histopathologic diagnosis of gastrointestinal disorders, namely, villus blunting, intraepithelial lymphocytes (IELs), goblet and Paneth cell depletion, and intramucosal Brunner’s glands. If >1 of these 5 parameters was not scorable because of technical issues, the TSP-5 was also considered nonscorable. When >1 slide image for an individual was available, the average scores across slides were used.

### Statistical analyses

We used univariate linear regression to measure associations between the enteropathogens and the 8 histopathology parameters and TSP-5 scores, although we previously hypothesized that pathogen detection would be associated with TSP-5 and 4 specific histology parameters based on their specific involvement in antimicrobial activity (Paneth cells) and mucin secretion (goblet cells) to protect against pathogens and inflammation (IELs and chronic inflammation in lamina propria). Datapoints recorded as not scorable were treated as missing and dropped from regression models. Unrounded mean histologic scores were modeled as outcomes. Pathogen detection (positive/negative) and burden (continuous) variables were modeled as predictors. Analyses were conducted for pathogens sought across all 3 centers and restricted to TAC results from BEED and SEEM. Although the pathogen burden was calculated for BEECH, it was not comparable with BEED and SEEM because the TAC platform included 18 additional organisms that were counted in this parameter. Hence, the pathogen burden and virulence locus (only available for TAC) burden were modeled as predictors in TAC-restricted analyses.

As this was an exploratory analysis, univariate relationships with a 2-tailed *P* value of <0.1 were further analyzed in multivariable models adjusting for the following covariates that were selected as potential confounders: age, study center, and time between sample collection and biopsy. A 2-tailed *P* value of <0.05 was considered statistically significant in multivariable models. Due to the novel and exploratory nature of this work and our modest sample size, we did not correct for multiple analyses, choosing to identify as many potential avenues for further study as possible. Analyses were conducted using the statistical software Stata/SE 16.1 and R (version 4.2.1).

### Ethical approvals

Signed informed consent from legal guardians was obtained and all study centers obtained approvals from their respective institutional ethical review boards. Details on these procedures and single-center cohort data relevant to this multicenter pathogen analysis that have been previously published can be found elsewhere in this supplemental issue [5]. Ethical approval was obtained from AKU Ethics Review Committee (ERC) (3836-Ped-ERC-15), icddr,b ERC (PR-16007), University of Zambia Biomedical Research Ethics Committee (006-02-16) and National Health Research Authority (MH/101/23/10/1), UVA Institutional Review Board (19466), CCHMC Institutional Review Board (2016-0387). Exemption was received from the University of Washington Institutional Review Board (IRB) (STUDY00013442) and the Washington University IRB (201801207).

## Results

**FIGURE 1:**
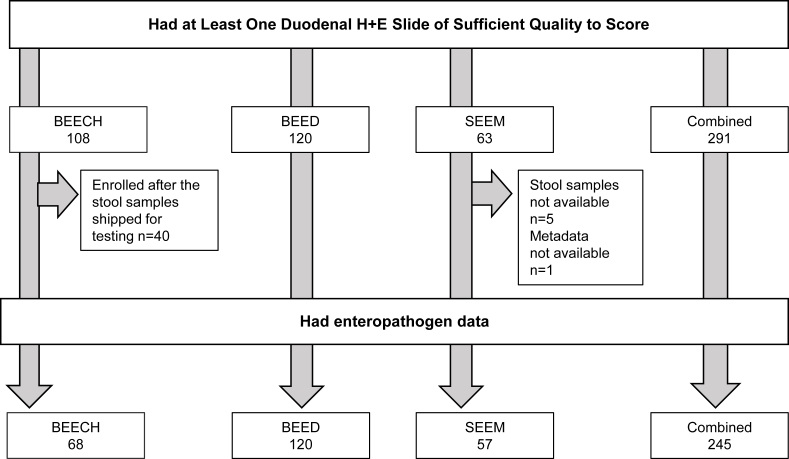
Enrollment flowchart. H&E, hematoxylin and eosin.

Of the 291 children with sufficient histology data, stool samples from 245 (84%) were evaluated for fecal enteropathogens and included in this analysis, including those from 68 BEECH, 120 BEED, and 57 SEEM participants (Figure 1).

### Demographic characteristics and fecal biopsy sampling

Demographic characteristics are presented in Table 1. Consistent with their respective recruitment strategies, more SEEM children and less BEED children were undernourished. The median (IQR) age at biopsy [1.6 (1.4, 1.8) y] was similar across the centers. The subgroup in this analysis resembles the larger cohort of all 291 children enrolled in the main analysis [5].

**TABLE 1:** Demographic characteristics by center

	BEECH *n* = 68	BEED *n* = 120	SEEM *n* = 57	All centers *n* = 245
Female, number (%)	36 (52.9)	70 (58.3)	17 (29.8)	123 (50.2)
LAZ^1^, median (IQR)	-3.3 (-4.0, -3.0)	-2.1 (-2.8, -1.5)	-3.2 (-3.7, -2.4)	-2.8 (-3.4, -2.0)
WAZ^1^, median (IQR)	-2.2 (-2.7, -1.8)	-1.7 (-2.3, -1.2)	-3.1 (-3.6, -2.6)	-2.2 (-2.8, -1.6)
WLZ^1^, median (IQR)	-0.8 (-1.3, -0.1)	-1.0 (-1.4, -0.4)	-2.2 (-2.8, -1.8)	-1.1 (-1.9, -0.5)
Age at stool sampling, y, median (IQR)	1.0 (0.8, 1.4)	1.2 (1.1, 1.4)	0.8 (0.8, 0.8)	1.1 (0.8, 1.3)
Age at biopsy, y, median (IQR)	1.6 (1.3, 1.8)	1.6 (1.4, 1.7)	1.7 (1.3, 1.8)	1.6 (1.4, 1.8)
Stool sampling to biopsy interval, mo, median (IQR)	4.4 (2.6, 6.9)	4.1 (3.9, 4.5)	11.3 (5.9, 12.7)	4.4 (3.9, 6.5)

Abbreviations: BEECH, Biomarkers of Environmental Enteropathy in Children; BEED, Bangladesh Environmental Enteric Dysfunction; LAZ, length-for-age *z*-score; SEEM, Study of Environmental Enteropathy and Malnutrition; WAZ, weight-for-age *z*-score; WLZ, weight-for-length *z*-score.

1 Measurements closest to the time of biopsy. Recumbent length is measured for children <2 y of age, while standing height is measured for those ≥ 2y of age. 8 children in the BEECH analysis were 2y of age at the anthropometric measurement closest to the time of biopsy.

The median (IQR) age at fecal sample collection was 1.2 (1.1, 1.4), 1.0 (0.8, 1.4), and 0.8 (0.8, 0.8) y for BEED, BEECH, and SEEM, respectively, consistent with each center’s study design [5]. Median intervals between stool collection and biopsy were similar for BEECH and BEED but longer for SEEM (4.4, 4.1, and 11.3 mo, respectively), again reflecting the study design of individual centers.

### Histology parameters

Histology scores in the subgroup with pathogen data (Table 2) resembled those from the entire Consortium cohort [6]. The TSP-5 was highest in BEED and lowest in SEEM children, whereas more severe Paneth and goblet cell depletion, intraepithelial lymphocytosis, and villus blunting were seen in the BEED, SEEM, and BEECH cohorts, respectively, as in the larger cohort.

**TABLE 2:** Histology scores by center^1^

	BEECH *n* = 68	BEED *n* = 120	SEEM *n* = 57	Sites combined *n* = 245
TSP-5 (0%–100%), median (IQR) Percent not scorable	52.8 (46.4, 61.1) 4.4%	58.6 (47.2, 66.7) 33.3%	46.4 (37.2, 52.8) 0%	52.5 (44.2, 61.1) 17.6%
Goblet cell depletion (0–4), median (IQR) Percent not scorable	1.5 (1.0, 2.0) 2.9%	2.0 (1.4, 2.5) 0%	1.0 (0.8, 1.3) 0%	1.5 (1.0, 2.0) 0.8%
Intraepithelial lymphocytes (0–4), median (IQR) Percent not scorable	1.0 (0.5, 1.5) 1.5%	1.5 (1.0, 2.0) 0.8%	1.7 (1.2, 2.5) 0%	1.5 (1.0, 2.0) 0.8%
Intramucosal Brunner glands (0–3), median (IQR) Percent not scorable	0.0 (0.0, 0.0) 1.5%	0.0 (0.0, 0.5) 3.3%	0.2 (0.0, 0.9) 3.5%	0.0 (0.0, 0.5) 2.9%
Paneth cell depletion (0–3), median (IQR) Percent not scorable	2.0 (1.0, 3.0) 17.6%	3.0 (1.9, 3.0) 46.7%	0.7 (0.4, 1.0) 15.8%	1.6 (1.0, 3.0) 31.4%
Villus architecture (0–4), median (IQR) Percent not scorable	2.5 (1.5, 3.5) 8.8%	2.0 (1.0, 3.0) 39.2%	2.0 (1.3, 3.0) 24.6%	2.0 (1.5, 3.2) 27.3%
Chronic inflammation (0–3), median (IQR) Percent not scorable	1.5 (1.2, 2.0) 2.9%	1.5 (1.0, 1.5) 1.7%	1.2 (1.0, 1.5) 1.8%	1.5 (1.0, 1.7) 2%
Enterocyte injury (0–3), median (IQR) Percent not scorable	0.2 (0.0, 0.5) 1.5%	0.3 (0.0, 0.5) 0%	0.4 (0.2, 0.5) 0%	0.3 (0.0, 0.5) 0.4%
Epithelial detachment (0–4), median (IQR) Percent not scorable	1.0 (0.6, 1.0) 0%	1.0 (0.5, 1.0) 0%	1.0 (0.8, 1.2) 0%	1.0 (0.5, 1.1) 0%

Abbreviations: BEECH, Biomarkers of Environmental Enteropathy in Children; BEED, Bangladesh Environmental Enteric Dysfunction; SEEM, Study of Environmental Enteropathy and Malnutrition, TSP-5, Total Score Percent 5.

1 For each histology parameter and the summative TSP-5, the possible range of scores is provided in the first column. The percent not scorable represents the proportion of slide images that were determined to be not scorable due to technical slide quality issues by the scoring pathologists.

### Enteropathogen prevalence

The preponderance of stools (97%) contained >1 enteric pathogen; 95%, 48%, 45%, and 3.4% contained a bacterial, protozoal, viral, or helminthic enteric pathogen, respectively (Table 3). The mean pathogen burden, defined as the number of pathogens per stool sample, was 4 across all centers.

**TABLE 3:** Enteropathogenic prevalence by center. N (%) unless otherwise noted

Enteropathogens	BEECH *n* = 68	BEED *n* = 120	SEEM *n* = 57	Overall *n* = 245^1^
Bacteria				
*Aeromonas*	NA	10 (8.3)	2 (3.5)	12 (6.8)
*Clostridioides difficile*	2 (2.9)	14 (11.7)	8 (14.0)	24 (9.8)
*Campylobacter*^2^	44 (64.7)	64 (53.3)	38 (66.7)	146 (59.6)
*Campylobacter,* pan	NA	62 (51.7)	37 (64.9)	99 (55.9)
*C. jejuni* or *coli*	NA	46 (38.3)	27 (48.2)	73 (41.5)
EAEC^3^	NA	80 (66.7)	49 (86)	129 (72.9)
EAEC, *aaiC*	NA	35 (29.4)^4^	20 (35.1)	55 (31.3)
EAEC, *aatA*	NA	69 (57)^4^	40 (70.2)	109 (61.9)
EPEC	NA	67 (55.8)	29 (50.9)	96 (54.2)
Typical EPEC^5^	NA	19 (15.8)	15 (26.3)	34 (19.2)
Atypical EPEC^5^	NA	48 (40)	14 (24.6)	62 (35)
ETEC	48 (70.6)	51 (42.5)	22 (38.6)	121 (49.4)
ETEC, heat-labile	NA	18 (15)	15 (26.3)	33 (18.6)
ETEC, heat stable^6^	NA	33 (27.5)	12 (21.1)	45 (25.4)
ETEC, heat stable, STh	NA	20 (16.8)^4^	9 (15.8)	29 (16.4)
ETEC, heat stable, STp	NA	14 (11.8)^4^	5 (8.8)	19 (10.8)
*Helicobacter pylori*	NA	5 (4.2)	1 (1.8)	6 (3.4)
*Plesiomonas shigelloides*	NA	8 (6.7)	0 (0)	8 (4.5)
*Salmonella enterica*	41 (60.3)	1 (0.8)	0 (0)	42 (17.1)
*Shigella*_EIEC	42 (61.8)	29 (24.2)	7 (12.3)	78 (31.84)
STEC	5 (7.4)	3 (2.5)	7 (12.3)	15 (6.1)
STEC, *stx1*	NA	3 (2.5)^4^	6 (10.5)	9 (5.1)
STEC, *stx2*	NA	0 (0)	2 (3.5)	2 (1.1)
*Vibrio cholerae*	1 (1.5)	1 (0.8)	2 (3.5)	4 (1.6)
Any bacterial pathogen	64 (94.1)	114 (95)	54 (94.7)	232 (94.7)
Viruses				
Adenovirus 40/41	9 (13.2)	10 (8.3)	12 (21.1)	31 (12.7)
Astrovirus	NA	5 (4.2)^7^	5 (8.8)	10 (5.7)
Norovirus	39 (57.3)	26 (21.7)^8^	19 (33.3)	45 (25.9)
Norovirus GI	NA	6 (5.1)^7^	6 (11.1)	12 (6.9)
Norovirus GII	NA	23 (19.5)^7^	15 (26.3)	38 (21.7)
Rotavirus	16 (23.5)	3 (2.5)^7^	3 (5.3)	22 (9.1)
Sapovirus^9^	NA	19 (16)^4^	15 (26.3)	34 (19.3)
Any viral pathogen	42 (61.8)	34 (28.3)	35 (61.4)	111 (45.3)
Protozoa				
*Cryptosporidium*	19 (27.9)	13 (10.8)	15 (26.3)	47 (19.2)
*Cylcospora cayetanensis*	NA	0 (0)	0 (0)	0 (0)
*Entamoeba*	1 (1.5)	1 (0.8)	NA	2 (1.1)
*E. histolytica*	NA	0 (0)	0 (0)	0 (0)
*Enterocytozoon bieneusi*	NA	12 (10)	5 (8.8)	17 (9.6)
*Encephalitozoon intestinalis*	NA	2 (1.7)	0 (0)	2 (1.1)
*Giardia lamblia*	47 (69.1)	NA^10^	41 (71.9)	88 (70.4)
*Cystoisospora belli*	NA	1 (0.8)	0 (0)	1 (0.6)
Any protozoal pathogen	49 (72.1)	25 (20.8)	44 (77.2)	118 (48.2)
Helminths				
*Ancyclostoma duodenale*	NA	0 (0)	0 (0)	0 (0)
*Ascaris lumbricoides*	NA	3 (2.5)	0 (0)	3 (1.7)
*Necator*	NA	0 (0)	0 (0)	0 (0)
*Strongyloides*	NA	0 (0)	0 (0)	0 (0)
*Trichuris*	NA	4 (3.3)	0 (0)	4 (2.3)
Any helminthic pathogen	NA	6 (5)	0 (0)	6 (3.4)
Any pathogen	67 (98.5)	115 (95.8)	56 (98.2)	238 (97.1)
Pathogen burden mean (SD, range)	4.0 (2.1, 0-9)	3.6 (1.8, 0-8)	4.9 (1.8, 0-9)	4.0 (2.0, 0-9)

Abbreviations: BEECH, Biomarkers of Environmental Enteropathy in Children; BEED, Bangladesh Environmental Enteric Dysfunction; EAEC, enteroaggregative *E. coli*; EIEC, enteroinvasive *E. coli*; EPEC, enteropathogenic *E. coli*; ETEC, enterotoxigenic *E. coli*; NA, data not available; SEEM, Study of Environmental Enteropathy and Malnutrition; STEC, Shiga toxin producing *E. coli*.

1 Among cohorts and patients with data.

2 For BEED and SEEM, which used TAC, this variable is defined as having gene targets cpn60 (pan *Campylobacter*) or *cadF* (*C. coli* or *C. jejuni*) positive.

3 For BEED and SEEM, which used TAC, this variable is defined as having targets *aaiC* or *aatA* positive.

4 *N* = 119 for BEED due to *n* = 1 missing data.

5 Typical EPEC is defined as positive for both targets *eae* and *bfpA*, whereas atypical EPEC is defined as positive for *eae* and negative for *bfpA*. Target prevalences were as follows: *eae:* BEED *n* = 1 missing, *n* = 69 (58%) positive, SEEM 29 (50.9%) positive; *bfpA* BEED *n* = 1 missing, *n* = 19 (16%) positive, SEEM 35 (20%) positive.

6 For BEED and SEEM, which used TAC, this variable is defined as positive for genes encoding STp or STh.

7 *N* = 118 for BEED due to *n* = 2 missing data.

8 *N* = 117 for BEED due to *n* = 3 missing data.

9 Genotypes I, II, or IV.

10 This target did not perform well on the BEED TAC cards.

### Bacterial enteropathogens

*Campylobacter*, enterotoxigenic *Escherichia coli* (ETEC), and *Shigella* were the most prevalent bacteria sought across all 3 centers. Approximately two-thirds of the SEEM (66.7%) and BEECH (64.7%) and half of the BEED (53.3%) cohorts were positive for *Campylobacter*. ETEC was highly prevalent, although to varying degrees across the cohorts: BEECH (71%), BEED (43%), and SEEM (39%). The overall prevalence of *Shigella* was 32%, although it was most commonly identified in BEECH (62%) and least often identified in SEEM (12%). BEECH had a high prevalence of *Salmonella* (60%), whereas BEED and SEEM samples were infrequently positive for this agent. Enteroaggregative *E. coli* (EAEC) and enteropathogenic *E. coli* (EPEC) targets were included only in the TAC platform. EAEC was identified in nearly three-quarters of stools, more commonly in SEEM (86%) than in BEED (67%), with the *aatA* locus being more prevalent in both cohorts. The prevalence of EPEC was similar across all cohorts (54% overall); each of atypical and typical EPEC was detected in one-quarter of SEEM stools, whereas atypical EPEC was >2-fold more prevalent in the BEED stools (40%) compared with typical EPEC (16%).

### Viral pathogens

Norovirus was the most commonly detected virus across all 3 centers (26%), with the highest prevalence in BEECH (57%), followed by SEEM (33%) and BEED (22%). TAC included genotype (G)/serotype specifications; GII was the predominant genotype in SEEM (26%) and BEED (20%). The detection of adenovirus 40/41 was the highest in SEEM (21%), followed by BEECH (13%) and BEED (8%). Rotavirus was detected in one-quarter of BEECH participants but much less frequently in SEEM (5%) and BEED (3%). During the study interval, rotavirus vaccination was not part of the Bangladeshi or Pakistani routine vaccine schedule, although it was administered as part of the SEEM protocol before fecal sample collection. It is part of the Zambian routine vaccine schedule; 97% of BEECH children in this analysis were vaccinated against rotavirus.

### Protozoal pathogens

*Cryptosporidium* was the only parasite sought across all 3 centers; its detection prevalence was similar in BEECH (28%) and SEEM (26%) but lower in BEED (11%). Data on *Giardia* were available for BEECH and SEEM; it was detected in over two-thirds of stools from these cohorts (BEECH 69% and SEEM 72%).

### Regressions to identify the enteropathogenic association with histology in tri-center analyses

Univariate models explored associations between histology and enteropathogens identified across all 3 centers (including *Giardia* for which results were unavailable for the BEED cohort) if the pathogen had an overall prevalence of >3% (Table 3). *Campylobacter*, *Clostridioides difficile,* ETEC, *Salmonella*, Shiga toxin-producing *E. coli* (STEC), *Shigella,* adenovirus 40/41, norovirus, rotavirus, and *Cryptosporidium* were associated with histologic parameters at the *P* value of <0.1 level. They were further explored in multivariable models adjusted for age, stool-biopsy time interval, and study center (Supplemental Table 4; regressions with standardized β coefficients are also provided in Supplemental Table 5). In the multivariable analysis, *C. difficile, Salmonella,* STEC, adenovirus 40/41, and *Cryptosporidium* lost statistical association with histology at the *P* value of <0.05 level. *Campylobacter* and ETEC were associated with the TSP-5 [β 4.2; 95% confidence interval (CI) 0.8, 7.7 and β 3.9; 95% CI: 0.5, 7.3, respectively) (Table 4). *Campylobacter* was also associated with IELs [β 0.2 (95% CI: 0.0, 0.4)]. Norovirus and *Shigella* were associated with greater enterocyte injury (β 0.2; 95% CI: 0.1, 0.3 and β 0.2; 95% CI: 0.0, 0.3).

**TABLE 4:** Results of multivariable regression analysis at all geographic sites (BEECH, BEED, and SEEM)^1^

Pathogen	Histologic parameter (range of possible scores)	N	β coefficient (95% CI)
*Campylobacter*	TSP-5 (0%–100%)	202	4.2 (0.8, 7.7)
	IELs (0–4)	243	0.2 (0.0, 0.4)
ETEC	TSP-5 (0%–100%)	202	3.9 (0.5, 7.3)
*Shigella*	Enterocyte injury (0–3)	244	0.2 (0.0, 0.3)
*Norovirus*	Enterocyte injury (0–3)	241	0.2 (0.1, 0.3)

Abbreviations: BEECH, Biomarkers of Environmental Enteropathy in Children; BEED, Bangladesh Environmental Enteric Dysfunction; CI, confidence interval; ETEC, enterotoxigenic *E. coli*; IELs, intraepithelial lymphocytes; SEEM, Study of Environmental Enteropathy and Malnutrition; TSP-5, total score percent 5.

1 Multivariable models were adjusted for age, time difference between stool collection and biopsy, and study center. β coefficients can be interpreted as a higher (positive coefficient) or lower (negative coefficient) histology parameter score when a pathogen was detected or not detected. For example, the TSP-5 score was 4.2% (ranging from 0% to 100%) higher and the IEL score (ranging from 0 to 4) was 0.2 points higher when *Campylobacter* was detected compared with when it was not, with other predictors constant.

### Regressions to identify enteropathogenic association with histology: TAC-only sensitivity analyses

We explored pathogens and their subtypes in this BEED/SEEM sensitivity analysis as above and for those not available in the BEECH Luminex platform (Supplemental Tables 1 and 2). We excluded *Salmonella* from further analysis because its prevalence was <3% in these 2 centers (Table 3). Regression results (univariate and multivariable) are presented in Supplemental Table 6. We also present regressions with standardized β coefficients in Supplemental Table 7. Table 5 presents the multivariable results. Three associations found in the 3-center analysis were consistent in the 2-center TAC-only analysis. The association between norovirus and enterocyte injury (β 0.1; 95% CI: 0.0, 0.3) was corroborated; norovirus GII also showed this relationship (β 0.1; 95% CI: 0.0, 0.3) and an association with chronic inflammation (β 0.2; 95% CI: 0.0, 0.4). Norovirus and the GII genogroup were also associated with IELs (both βs 0.4; 95% CI: 0.1, 0.7 and 95% CI: 0.2, 0.7, respectively). The second consistent association between the 3- and 2-center analyses was between ETEC and TSP-5 (β 4.6; 95% CI: 0.1, 9.1). In the sensitivity analysis, ETEC (β 0.3; 95% CI: 0.1, 0.6), heat-stable ETEC (β 0.4; 95% CI: 0.1, 0.8), and the STh locus (β 0.4; 95% CI: 0.1, 0.7) were also associated with IELs. Although the association between *Campylobacter* and TSP-5 in the 3-center analysis did not hold in the 2-center analysis, IELs were associated with both *Campylobacter* TAC targets with equivalent point estimates and CIs (β 0.3; 95% CI: 0.0, 0.5).

**TABLE 5:** Results of multivariable regression from BEED and SEEM TAC data only^1^

Pathogen	Histologic **p**arameter (possible range of scores)	*N*	β coefficient	95% CI
*Aeromonas*	TSP-5 (0–100%)	137	8.1	0.5, 15.6
	Enterocyte injury (0–3)	177	0.4	0.2, 0.6
*Campylobacter*^2^	IELs (0–4)	176	0.3	0.0, 0.5
*Campylobacter*, pan	IELs (0–4)	176	0.3	0.0, 0.5
*C. jejuni* or *coli*	IELs (0–4)	175	0.3	0.0, 0.5
*Clostridioides difficile*	Enterocyte injury (0–3)	177	-0.2	-0.3, -0.0
Typical EPEC	Intramucosal Brunner’s glands (0–3)	171	-0.3	-0.6, -0.0
	IELs (0–4)	176	0.3	0.0, 0.7
ETEC	TSP-5 (0–100%)	137	4.6	0.1, 9.1
	IELs (0–4)	176	0.3	0.1, 0.6
ETEC, heat stable^3^	IELs (0–4)	176	0.4	0.1, 0.8
ETEC, heat stable, STh	IELs (0–4)	175	0.4	0.1, 0.7
*Plesiomonas shigelloides*	Epithelial detachment (0–4)	177	0.6	0.1, 1.0
Norovirus	Enterocyte injury (0–3)	174	0.1	0.0, 0.3
	IELs (0–4)	173	0.4	0.1, 0.7
Norovirus GII	Enterocyte injury (0–3)	175	0.1	0.0, 0.3
	Chronic inflammation (0–3)	172	0.2	0.0, 0.4
	IELs (0–4)	174	0.4	0.2, 0.7
*Enterocytozoon bienuesi*	IELs (0–4)	176	0.5	0.0, 0.9
Pathogen burden	Chronic inflammation (0–3)	174	0.0	0.0, 0.1
	Enterocyte injury (0–3)	177	0.0	0.0, 0.1
	IELs (0–4)	176	0.2	0.1, 0.2
Virulence locus burden	Chronic inflammation (0–3)	174	0.0	0.0, 0.1
	IELs (0–4)	176	0.1	0.1, 0.2
			

Abbreviations: BEED, Bangladesh Environmental Enteric Dysfunction; CI, confidence interval; ETEC, enterotoxigenic *E. coli*; IELs, intraepithelial lymphocytes; SEEM, Study of Environmental Enteropathy and Malnutrition; TAC, Taq-Man array card; TSP-5, total score percent-5.

1 Multivariable models were adjusted for age, time difference between stool collection and biopsy, and study center. β coefficients for individual pathogens can be interpreted as a higher (positive coefficient) or lower (negative coefficient) histology parameter score when a pathogen was detected or not detected. For example, the mean TSP-5 score was 8.1% (ranging from 0% to 100%) higher and the mean enterocyte injury score (ranging from 0 to 3) was 0.4 points higher when *Aeromonas* was detected compared with when it was not, with other predictors constant. β coefficients for pathogen burden variables represent mean differences in histology parameters with every unit higher in the burden variable. For example, the IELs score was 0.2 points higher with every additional pathogen detected.

2 Defined as targets *cpn60* (pan *Campylobacter*) or *cadF* (*C. coli* or *C. jejuni*) positive.

3 Defined as heat-stable genes encoding STp or STh positive.

*C. difficile* was inversely related to enterocyte injury in the 2-center analysis (β -0.2; 95% CI: -0.3, -0.0). Of the pathogens only assessable on the TAC platform, *Aeromonas,* typical EPEC, *P. shigelloides,* and *E. bienuesi* were significantly associated with histologic parameters (Table 5).

Pathogen burden and virulence locus burden were associated with IELs (β 0.2; 95% CI: 0.1, 0.2 and β 0.1; 95% CI: 0.1, 0.2, respectively). Pathogen burden was also associated with chronic inflammation and enterocyte injury, whereas virulence locus burden was associated with chronic inflammation, although the β coefficients were small (0.047, 0.008, and 0.038 respectively).

## Discussion

To our knowledge, this study is the first attempt to determine if fecal enteropathogenic carriage predicts small-bowel histopathology across 3 geographically distinct pediatric cohorts using a standardized and validated EED histology scoring system. Carriage without diarrhea is common among young children in low-resource settings, especially the undernourished ones [13,[16], [17], [18]]. Virtually all (97%) children in our 3-center cohort had >1 enteropathogen detected, including 95%, 48%, and 45% with a bacteria, protozoa, or virus, respectively, in the absence of diarrhea. Although it is not possible to directly compare carriage prevalence with other studies due to different platform contents, the prevalence in our study was similar to other studies. The enteropathogenic carriage was detected among 65% and 72% of similarly aged children without diarrhea in the 8-country community-based Malnutrition and Enteric Disease (MAL-ED) cohort and the 8-country case-control Global Enteric Multicenter Study (GEMS), respectively [19,20], whereas a South Indian cross-sectional comparison of asymptomatic infants and older children from economically disadvantaged and advantaged communities found enteropathogenic carriage of 93% and 72%, respectively [21]. Except for *Giardia*, the most prevalent pathogens in our cohort converged with the other reports and included *Campylobacter* spp., EAEC, EPEC, ETEC, *Shigella* spp., and norovirus. Pathogen communities, even in the absence of symptoms of gastroenteritis, i.e., the pathobiomes, have been postulated as a putative cause of EED, either directly affecting intestinal structures or indirectly by perturbing the gut microbiome, causing potential microbial invasion into the mucus or tissue [22].

The pathogens associated with subsequent histology in our 3-center analysis included norovirus and *Shigella* (with enterocyte injury), *Campylobacter* (with IELs and TSP-5), and ETEC (with TSP-5). To our knowledge, histologic data on subclinical infections or after pathogen detection are nonexistent, and the histopathology of symptomatic enteric infections has been described to variable extents among adults and rarely among children. *Shigella* multiplies in the small intestine and invades the large bowel causing enteritis characterized by the infiltration of epithelial cells with neutrophils and subsequent inflammatory pseudomembrane formation [23]. *Campylobacter* also primarily induces colitis with crypt architectural distortion and the loss of surface epithelium; variable inflammatory responses have been noted [24,25]. ETEC is among the few bacterial pathogens that infect the upper small bowel, adhering to enterocytes, and secreting heat-labile or heat-stable enterotoxins [25]. Norovirus also induces small-bowel enteritis, with intraepithelial lymphocytosis reported [26].

Our sensitivity analysis of TAC-generated data from the 2 South Asian centers allowed us to explore identical targets sought by the same technology. The analysis corroborated the association between *Campylobacter* and IELs, identified that 72% of *Campylobacter* detections were positive for the *C. coli/jejuni* (*cadF*) target, and demonstrated similar effect sizes for the relationship of IELs with the pan (*cpn60*) and *C. coli/jejuni* targets. This was somewhat unexpected as Francois et al. [27]. demonstrated that non-*C. coli/jejuni* were more common than *C. coli/jejuni* in the Peruvian 361 MAL-ED cohort and only the latter was associated with fecal myeloperoxidase, a marker of gut inflammation (although neutrophil derived). Our TAC-only analysis also corroborated the association between ETEC and TSP-5 and identified an association with IELs with specification to the STh heat-stable locus. The prediction of norovirus with enterocyte injury was corroborated, and an association with IELs emerged. These relationships, and an association with chronic inflammation, were found with the GII but not GI genotype. GII was also more prevalent, consistent with findings from other studies globally [28]. Sample size may have mitigated the association between *Shigella* and enterocyte injury in the TAC-only analysis, as this species was identified most frequently in the Zambian cohort. *C. difficile* was unexpectedly inversely associated with enterocyte injury in the 2-center analysis. However, toxigenic *C. difficile* is not a pathogen in children aged <2 y, and its pathogenicity during much of childhood is questionable [29,30].

The 2-site TAC-restricted analysis also allowed exploration of pathogens not included in the BEECH Luminex platform. An unexpected finding of typical EPEC predicting reduced intramucosal Brunner’s glands is difficult to explain. Brunner’s glands are normally submucosal, secreting alkaline mucus to neutralize gastric acid, and increased densities in the mucosa are not a feature of EED [6]. Typical EPEC was associated with IELs. In enteritis, EPEC adheres to small-bowel enterocytes and induces an inflammatory response, whereas both typical and atypical EPEC have been associated with reduced WAZ and WHZ in an analysis adjusted for diarrhea status [31,32]. *Aeromonas*, *P. shigelloides*, and *E. bieneusi* also showed associations with histologic abnormalities. *E. bieneusi* in non-diarrheal stools was associated with poor linear growth in subsequent months in MAL-ED, whereas *Aeromonas* was associated with mortality following moderate-to-severe diarrhea in GEMS [33,34].

As in other studies using highly sensitive PCR platforms, we identified a high prevalence of coinfection – a mean of 4.0 pathogens and 4.7 virulence loci per stool among those tested by TAC [21,28]. Praharaj et al. [21] found 3.3 enteropathogens per stool among children from an economically disadvantaged community in India. Although they found that stools with no pathogens had similar levels of fecal intestinal inflammatory markers as those with a single pathogen, stools with multiple pathogens showed elevated concentrations of myeloperoxidase and calprotectin. In our study, increasing pathogen burden and virulence locus burden were associated with higher IEL scores.

Besides the challenges in accounting for coinfections in the setting of comprehensive detection panels, our analysis was limited by differences in testing technologies, although we attempted to overcome this with a sensitivity analysis. Although we cannot rule out the possibility of reverse causality, the temporality of our exposure variable preceding the histology outcome measure by months reduces this probability. There was variability in the enrollment undernutrition criteria and stool collection intervals relative to biopsy, both inherent to dissimilarities in the 3 study designs. We attempted to overcome these by adjusting for stool-biopsy collection intervals and study centers and demonstrated geographic enteropathogenic variability resembling the findings of MAL-ED and GEMS [20,33]. We were limited to the subtype and serotype-level data available on the TAC platform which, when available, demonstrated important within-species differences in the associations between prevalence and histology. We were also limited to single stool and biopsy samples. As this was a novel opportunity to leverage biopsy and fecal enteropathogenic data, we assessed for relationships between histologic parameters and several enteropathogens, which increases the risk of false discovery. Pediatric endoscopy studies in low-resource settings have been limited due to the cost, technical expertise needed, and ethical considerations in extending the procedure beyond children who benefit clinically [35]. Enteroids and gut-on-a-chip technologies offer experimental opportunities, for example, to determine pathogen- or viral strain-specific enterocyte death in future studies [36,37].

The etiology of EED remains elusive. Despite the limitations, the EEDBI Consortium offers a novel opportunity to explore associations between fecal enteropathogenic detection in the absence of diarrhea and subsequent histopathologic features of EED using a validated histologic scoring system. Examining pathogen associations with the target organ – the small bowel – provides an unprecedented analysis of relationships between enteropathogens with EED histology features in children with EED across geographies.

## Acknowledgments

We thank Louise Warren for help in manuscript preparation.

## Author contributions

The authors’ responsibilities were as follows – NTI, TA, MMa, PK: designed the research; NTI, KC, SMF, TA, MMa, FK, PK: conducted the research; ERH, JL: provided the TaqMan array cards; SL, PIT, DMD: managed the data; SL, KV, MMw: analyzed the data; KV, PIT, DMD: interpreted the data; NTI: wrote the paper; PIT, DMD: edited the manuscript; NTI, DMD: had primary responsibility for the final content; and all authors: read and approved the final manuscript.

## Conflict of interest

PIT reports being a board member, receiving consultant/advisory fees, and having equity and stock ownership in MediBeacon, Inc. MediBeacon, Inc. has been issued patent on this technology, on which he is a co-inventor, and which might generate royalties. He is also a consultant to Temple University, is Chair of the Scientific Advisory Board of the American Gastroenterological Association Center for Microbiome Science and Education, and receives royalties from UpToDate for sections on *Escherichia coli* diarrhea. KV reports consulting income from Washington University in St. Louis. All other authors report no conflicts of interest.

## Funding

The EEDBI Consortium was funded by the following grants from the Bill and Melinda Gates Foundation: OPP1152812¸ OPP1066118, OPP1136759, OPP1138727, and OPP1144149, and the Advanced Imaging and Tissue Analysis Core of the Washington University Digestive Diseases Research Core Center P30DK052574.

## Data availability

Data described in the manuscript, code book, and analytic code will be made available upon request to the corresponding author pending application and approval.

## Figures and Tables

**Table:** 

Surname	Name	Full Affiliation	Email
Ahmed	Kumail	Department of Paediatrics and Child Health, Aga Khan University, Karachi, Pakistan	kumail.ahmed@aku.edu
Ahmed	Sheraz	Department of Paediatrics and Child Health, Aga Khan University, Karachi, Pakistan	sheraz.ahmed@aku.edu
Alam	Md Ashraful	Nutrition Research Division, International Centre for Diarrhoeal Disease Research, Bangladesh, Dhaka, Bangladesh	mashraful@icddrb.org
Ali	S Asad	Department of Paediatrics and Child Health, Aga Khan University, Karachi, Pakistan	asad.ali@aku.edu
Amadi	Beatrice	Tropical Gastroenterology and Nutrition Group, University of Zambia, Lusaka, Zambia	beatriceamadi@ymail.com
Das	Subhasish	Nutrition Research Division, International Centre for Diarrhoeal Disease Research, Bangladesh, Dhaka, Bangladesh	subhasish.das@icddrb.org
Gazi	Md Amran	Nutrition Research Division, International Centre for Diarrhoeal Disease Research, Bangladesh, Dhaka, Bangladesh	amran.gazi@icddrb.org
Haque	Rashidul	Infectious Disease Division, International Centre for Diarrhoeal Disease Research, Bangladesh, Dhaka, Bangladesh	rhaque@icddrb.org
Hasan	Md Mehedi	Nutrition Research Division, International Centre for Diarrhoeal Disease Research, Bangladesh, Dhaka, Bangladesh	md.hasan@icddrb.org
Hossain	Md Shabab	Nutrition Research Division, International Centre for Diarrhoeal Disease Research, Bangladesh, Dhaka, Bangladesh	dr.shabab@icddrb.org
Hotwani	Aneeta	Department of Paediatrics and Child Health, Aga Khan University, Karachi, Pakistan	aneeta.hotwani@aku.edu
Hussain	Shahneel	Department of Biological and Biomedical Sciences, Aga Khan University, Karachi, Pakistan	shahneel.hussain@aku.edu
Iqbal	Junaid	Department of Paediatrics and Child Health, Aga Khan University, Karachi, Pakistan	junaid.iqbal@aku.edu
Jakhro	Sadaf	Department of Paediatrics and Child Health, Aga Khan University, Karachi, Pakistan	sadaf.jakhro@aku.edu
Liu	Ta-Chiang	Department of Pathology and Immunology, Washington University, St. Louis, MO, USA	ta-chiang.liu@wustl.edu
Mazumder	Ramendra Nath	Nutrition Research Division, International Centre for Diarrhoeal Disease Research, Bangladesh, Dhaka, Bangladesh	ramen@icddrb.org
Moskaluk	Christopher A	Department of Pathology, University of Virginia, Charlottesville, VA, USA	cam5p@virginia.edu
Qureshi	Abdul Khalique	Department of Paediatrics and Child Health, Aga Khan University, Karachi, Pakistan	abdul.qureshi@aku.edu
Raghavan	Shyam S	Department of Pathology, University of Virginia, Charlottesville, VA, USA	SR7WQ@hscmail.mcc.virginia.edu
Rahman	Masudur	Department of Gastroenterology, Sheikh Russel National Gastroliver Institute and Hospital, Dhaka, Bangladesh	drmasud47@yahoo.com
Rahman	Najeeb	Department of Paediatrics and Child Health, Aga Khan University, Karachi, Pakistan	najeeb.rahman@aku.edu
Sadiq	Kamran	Department of Paediatrics and Child Health, Aga Khan University, Karachi, Pakistan	kamran.sadiq@aku.edu
Sarker	Shafiqul Alam	Nutrition Research Division, International Centre for Diarrhoeal Disease Research, Bangladesh, Dhaka, Bangladesh	sasarker@icddrb.org
Sullivan	Peter B	Department of Paediatrics, Children's Hospital, University of Oxford, Oxford, UK	peter.sullivan@paediatrics.ox.ac.uk
Tearney	Guillermo J	Department of Pathology, Harvard Medical School, Boston, MA, USA	gtearney@partners.org
Umrani	Fayaz	Department of Paediatrics and Child Health, Aga Khan University, Karachi, Pakistan	fayaz.umrani@aku.edu
Yilmaz	Omer H	Department of Pathology, Massachusetts General Hospital, Boston, MA, USA	oyilmaz@mgh.harvard.edu
Zyambo	Kanekwa	Tropical Gastroenterology & Nutrition Group, University of Zambia, Lusaka, Zambia	kanekwa@tropgan.net
