# Supplementary material for: Enteric pathogens relationship with small bowel histologic features of environmental enteric dysfunction in a multicountry cohort study
Source: Am J Clin Nutr. 2024 Sep 17;120(Suppl 1):S84–93. doi: 10.1016/j.ajcnut.2024.02.026 (PMC13168960; doi:10.1016/j.ajcnut.2024.02.026)
Supplement: Multimedia component 1 [file mmc1.zip › ajcnut_468_NAJEEH~1_mmc1.DOC]

**Supplemental Table 1. Enteropathogen targets in BEED and SEEM TAC cards**

| Enteropathogen targets | Locus or protein encoded^1^ | |
| --- | --- | --- |
|  | SEEM and BEED | BEECH |
| *Aeromonas* | Aerolysin |  |
| *Clostridioides difficile* | *tcdA* and *tcdB* | Included |
| *Campylobacter*_pan | *cpn60* | *Campylobacter* NOS included |
| *Campylobacter* *jejuni/C.* *coli* | *cadF* |  |
| EAEC (Enteroaggregative *E. coli*)_aaiC | *aaiC* |  |
| EAEC (Enteroaggregative *E. coli*)_aatA | *aatA* |  |
| EPEC (Enteropathogenic *E. coli*)_bfpA | *bfpA* |  |
| EPEC (Enteropathogenic *E. coli*)_eae | *eae* |  |
| ETEC (Enterotoxigenic *E. coli*)_LT | LT | ETEC NOS included  ETEC NOS included  ETEC NOS included |
| ETEC (Enterotoxigenic *E. coli*)_STh | STh |  |
| ETEC (Enterotoxigenic *E. coli*)_STp | STp |  |
| *Helicobacter pylori* | *ureC* |  |
| *Plesiomonas shigelloides* | *gyrB* |  |
| *Salmonella enterica* | *ttr* | Included |
| *Shigella*_EIEC (Enteroinvasive *E. coli*) | *ipaH* | Included |
| STEC (Shiga toxin-producing *E. coli*)_stx1 | *stx1* | STEC NOS included  STEC NOS included |
| STEC (Shiga toxin-producing *E. coli*)_stx2 | *stx2* |  |
| *Vibrio cholerae* | *hlyA* | Included |
| Adenovirus 40/41 | Fiber gene | Included |
| Astrovirus | Capsid |  |
| Norovirus GI | *ORF1-2* | Norovirus NOS included |
| Norovirus GII | *ORF1-2* |  |
| Rotavirus | *NSP3* | Included |
| Sapovirus (genotypes I-II-IV) | *RdRp* |  |
| *Cryptosporidium* | 18S rRNA | Included |
| *Cylcospora cayetanensis* | 18S rRNA |  |
| *Entamoeba* | 18S rRNA | Included |
| *Entamoeba histolytica* | 18S rRNA |  |
| *Enterocytozoon bieneusi* | *ITS* |  |
| *Encephalitozoon intestinalis* | SSU rRNA |  |
| *Giardia lamblia* | 18S rRNA | Included |
| *Cystoisospora belli* | 18S rRNA |  |
| *Ancylostoma duodenale* | *ITS2* |  |
| *Ascaris lumbricoides* | *ITS1* |  |
| *Necator americanus* | *ITS2* |  |
| *Strongyloides stercoralis* | Dispersed repetitive sequence |  |
| *Trichuris trichiura* | 18S rRNA |  |

^1^ Loci information proprietary to Luminex. All targets listed above (except *bfpA*) were counted separately in defining the virulence locus burden that was restricted to SEEM and BEED data only.

Abbreviations: BEECH, Biomarkers of Environmental Enteropathy in Children; BEED, Bangladesh Environmental Enteric Dysfunction; NOS, not otherwise specified; SEEM, Study of Environmental Enteropathy and Malnutrition; TAC, TaqMan array card

**Supplemental Table 2. Definition of pathogen burden.**

All pathogens listed in the left column were available for SEEM and BEED except for *Giardia* which was not available for BEED. Each of the pathogens were counted as a detected pathogen if determined positive in the TAC analysis. Notes regarding how multiple TAC loci are used to define a single pathogen are included in the middle column. Targets included in the BEECH Luminex platform are proprietary; Luminex-defined pathogens are included in the right column. Each of these were counted as a detected pathogen if determined positive in the Luminex analysis.

| Enteropathogens | SEEM and BEED | BEECH |
| --- | --- | --- |
|  |  |  |
| Bacteria | n=12 | n=7 |
| *Aeromonas* |  |  |
| *Clostridioides difficile* | *tcdA* or *tcdB* positive | Included |
| *Campylobacter*^1^ | *cpn60* or *cadF* positive | Included |
| Enteroaggregative *E. coli* (EAEC)^2^ | *aaiC* or *aatA* positive |  |
| Enteropathogenic *E. coli* (EPEC)^3^ | *eae* positive |  |
| Enterotoxigenic *E. coli* (ETEC)^4^ | LT, STh, or STp positive | Included |
| *Helicobacter pylori* |  |  |
| *Plesiomonas shigelloides* |  |  |
| *Salmonella enterica* |  | Included |
| *Shigella*_EIEC | *ipaH* positive^5^ | Included |
| Shiga toxin-producing *E. coli* (STEC)^6^ | *stx1* or *stx2* positive | Included |
| *Vibrio cholerae* |  | Included |
| Viruses | n=6 | n=3 |
| Adenovirus 40/41 |  | Included |
| Astrovirus |  |  |
| Norovirus GI^7^ |  | Norovirus NOS |
| Norovirus GII^7^ |  |  |
| Rotavirus |  | Included |
| Sapovirus (genotypes I-II-IV) |  |  |
| Protozoa | n=8 | n=3 |
| *Cryptosporidium* |  | Included |
| *Cylcospora cayetanensis* |  |  |
| *Entamoeba* |  | Included |
| *E. histolytica* |  |  |
| *Enterocytozoon bieneusi* |  |  |
| *Encephalitozoon intestinalis* |  |  |
| *Giardia lamblia* |  | Included |
| *Cystoisospora belli* |  |  |
| Helminths | n=5 | n=0 |
| *Ancyclostoma duodenale* |  |  |
| *Ascaris lumbricoides* |  |  |
| *Necator* |  |  |
| *Strongyloides* |  |  |
| *Trichuris* |  |  |
| Pathogen | n=31 | n=13 |

^1^ Any *Campylobacter* was defined as Luminex positive or TAC pan (*cpn60*) or jejuni_coli (*cadF*) positive and was counted as a single pathogen in the pathogen burden analysis while *cpn60* and *cadF* were each counted separately in the virulence locus burden analysis restricted to BEED and SEEM TAC only.

^2^ Any EAEC was defined as TAC *aaiC* or *aatA* positive and was counted as a single pathogen in the pathogen burden analysis while *aaiC* and *aaTA* were each counted separately in the virulence locus burden analysis restricted to BEED and SEEM TAC only.

^3^ Typical EPEC is defined as both TAC *eae* and *bfpA* positive while atypical EPEC is defined as positive *eae* and negative *bfpA.* Any EPEC is defined as the presence of typical or atypical EPEC (i.e., positive for *eae* regardless of *bfpA* status) and was counted as a single pathogen in the pathogen burden analysis and was counted as a single virulence locus in that burden analysis (restricted to SEEM and BEED TAC only)*.*

^4^ Heat labile ETEC is defined as LT positive while heat stable ETEC is defined as having STp or STh positive. Any ETEC is defined as Luminex positive or the presence of heat labile or heat stable ETEC for BEED and SEEM which used TAC. Any ETEC was counted as a single pathogen in the pathogen burden analysis while LT, STp, and STh were each counted separately in the virulence locus burden analysis restricted to BEED and SEEM TAC only.

^5^ *ipaH* detects both *Shigella* and enteroinvasive *E. coli,* however, based on previous studies demonstrating the majority of *ipaH* detections were for *Shigella,* we consider *ipaH* as indicative of *Shigella.*

^6^ Any STEC is defined as Luminex positive or the presence of *stx1* or *stx2* for BEED and SEEM which used TAC. Any STEC was counted as a single pathogen in the pathogen burden analysis while *stx1* and *stx2* were each counted separately in the virulence locus burden analysis restricted to BEED and SEEM TAC only.

^7^ Any norovirus is defined as Luminex positive or TAC GI or GII positive. However, norovirus GI and GII were counted separately in the pathogen burden analysis as previous studies have demonstrated that these are likely discrete pathogens. Only one variable (norovirus) was available in the Luminex platform; hence this was counted as a single pathogen in the pathogen burden analysis. GI and GII were also counted separately in the virulence locus burden analysis restricted to BEED and SEEM TAC only.

Abbreviations: BEECH, Biomarkers of Environmental Enteropathy in Children; BEED, Bangladesh Environmental Enteric Dysfunction; SEEM, Study of Environmental Enteropathy and Malnutrition

**Supplemental Table 3. Description of histology parameter scoring.**

| Goblet cell density depletion^1^ | 0: Normal goblet cell density (at least 1 goblet cell per 20 enterocytes) in all evaluable mucosal epithelial layer | 1: Decreased goblet cells (<1/20 enterocytes) in 1-25% of evaluable mucosal epithelium | 2: Decreased goblet cells (<1/20 enterocytes) in 26-50% of evaluable mucosal epithelium | 3: Decreased goblet cells (<1/20 enterocytes) in 51-75% of evaluable mucosal epithelium | 4: Decreased goblet cells (<1/20 enterocytes) in 76-100% of evaluable mucosal epithelium | NS: Not scorable |
| --- | --- | --- | --- | --- | --- | --- |
| Intra  epithelial lymphocytes^1^ | 0: No areas observed with epithelial/lymphocyte ratio >20% | 1: Lymphocyte/ epithelial ratio >20%, but <50%, in less than 50% of mucosa | 2: Lymphocyte/ epithelial ratio >20%, but <50%, in greater than 50% of mucosa | 3: Lymphocyte/ epithelial ratio >50% in less than 50% of mucosa | 4: Lymphocyte/ epithelial ratio >50% in greater than 50% of mucosa | NS: Not scorable |
| Intramucosal Brunner’s glands^1^ | 0: None observed | 1: One or two foci of intramucosal Brunner glands, none involving more than 5 crypt bases | 2: 3-5 foci of intramucosal Brunner glands, none involving more than 5 crypt bases | 3: > 5 foci, or any area of intramucosal Brunner glands involving >5 crypt bases |  | NS: Not scorable |
| Paneth cell density depletion^1^ | 0: >5 Paneth cells/ crypt base, on average | 1: 2-4 Paneth cells/ crypt base, on average | 2: <2 Paneth cell/crypt base, involving <50% of crypt bases | 3: <2 Paneth cell/crypt, involving >50% of crypt bases |  | NS: Not scorable |
| Villus architecture^1^ | 0: Majority of villi are >3 crypt lengths long | 1: Villi are < 3 but > 1 crypt lengths long, with abnormality involving ≤ 50% of mucosa | 2: Villi are < 3 but > 1 crypt lengths long, with abnormality involving > 50% of mucosa | 3: Villi absent, or <1 crypt length long, with abnormality involving ≤ 50% of mucosa | 4: Villi absent, or <1 crypt length long, with abnormality involving > 50% of mucosa | NS: Not scorable |
| Chronic inflammation | 0: No qualitative increase in mononuclear inflammatory cells (MIC) in lamina propria. Majority of villus bases contain <3 MIC across, on average | 1: Increased MIC, based on villus base displaying 3-5 MIC across, on average | 2: Increased MIC, based on villus base displaying 6-10 MIC across, on average | 3: Increased MIC, based on villus base displaying >10 lymphocytes on average |  | NS: Not scorable |
| Enterocyte injury | 0: Majority of enterocytes (90%) show tall columnar morphology | 1: Enterocytes show low columnar (<2:1 L:W ratio), cuboidal or flat morphology, in ≤ 50% of mucosa | 2: Enterocytes show low columnar (<2:1 L:W ratio), cuboidal or flat morphology, in > 50% of mucosa | 3: Any area of mucosal erosion/ulceration |  | NS: Not scorable factor |
| Epithelial detachment | 0: Complete coverage of mucosal surface by epithelial cells | 1: Surface epithelium missing or detached from <25% of mucosa | 2: Surface epithelium missing or detached from 25-50% of mucosa | 3: Surface epithelium missing or detached from 51-75% of mucosa | 4: Surface epithelium missing or detached from >75% of mucosa | NS: Not scorable |

**^1^**These five histology parameters are included in the calculation of the Total Score Percent-5 (TSP-5). At least four of these five parameters must have a numeric score (i.e., not non-scorable) for a slide to have a TSP-5 calculated. The numerator of the TSP-5 is the sum of the score of the scorable parameters and the denominator is the sum of the possible maximum score of the scorable parameters. Intramucosal Brunner’s glands are reversed coded when included in the TSP-5 due to their inverse relationship with EED compared to a comparison group without gastrointestinal histopathology or clinical diagnosis.

Abbreviations: NS, not scorable; variable cannot be determined because of slide quality or other factor

**Supplemental Table 4. Regression results, BEECH, BEED, and SEEM.**

|  |  |  | Univariate | | Multivariable | |
| --- | --- | --- | --- | --- | --- | --- |
| Pathogens | Histologic Parameters | N | β coefficient | 95% CI | β coefficient | 95% CI |
| *Campylobacter* | TSP-5 (0-100%) | 202 | 3.2 | -0.5, 6.9 | 4.2 | 0.8,7.7 |
|  | Chronic inflammation (0-3) | 240 | 0.1 | -0.0, 0.3 | 0.1 | -0.0, 0.3 |
|  | IELs (0-4) | 243 | 0.2 | -0.0, 0.4 | 0.2 | 0.0, 0.4 |
| *Clostridioides difficile* | TSP-5 (0-100%) | 202 | -6.7 | -13.1, -0.4 | -5.6 | -11.5, 0.4 |
|  | Enterocyte injury (0-3) | 244 | -0.2 | -0.3 ,0.0 | -0.2 | -0.3, 0.0 |
|  | VA (0-4) | 178 | -0.6 | -1.3, -0.0 | -0.5 | -1.2, 0.1 |
| *Enterotoxigenic E. coli* (ETEC) | TSP-5 (0-100%) | 202 | 4.2 | 0.6, 7.8 | 3.9 | 0.5, 7.3 |
|  | Chronic inflammation (0-3) | 240 | 0.2 | 0.0, 0.3 | 0.1 | -0.0, 0.3 |
|  | Intramucosal Brunner’s glands | 238 | -0.2 | -0.3, 0.0 | -0.1 | -0.3, 0.1 |
|  | Paneth cell depletion (0-3) | 168 | 0.4 | 0.1, 0.7 | 0.3 | -0.0, 0.5 |
| *Salmonella* | Chronic inflammation (0-3) | 240 | 0.3 | 0.1, 0.4 | 0.1 | -0.2, 0.4 |
|  | IELs (0-4) | 243 | -0.6 | -0.9, -0.4 | -0.3 | -0.6, 0.1 |
|  | Goblet cell depletion (0-4) | 243 | -0.2 | -0.5, 0.0 | -0.2 | -0.5, 0.2 |
|  | Paneth cell depletion (0-3) | 168 | 0.4 | 0.0, 0.8 | 0.2 | -0.3, 0.6 |
| Shiga toxin producing *E. coli* | Goblet cell depletion (0-4) | 243 | -0.5 | -0.9, -0.1 | -0.3 | -0.6, 0.1 |
| *Shigella* | TSP-5 (0-100%) | 202 | 4.4 | 0.6, 8.3 | 3.7 | -0.3, 7.7 |
|  | Chronic inflammation (0-3) | 240 | 0.2 | 0.0, 0.4 | 0.1 | -0.0, 0.3 |
|  | Enterocyte injury (0-3) | 244 | 0.1 | 0.0, 0.2 | 0.2 | 0.0, 0.3 |
|  | Paneth cell depletion (0-3) | 168 | 0.5 | 0.1, 0.8 | 0.2 | -0.1, 0.5 |
| Adenovirus 40/41 | Chronic inflammation (0-3) | 240 | 0.2 | -0.0, 0.4 | 0.2 | -0.0, 0.4 |
|  | Intramucosal Brunner’s glands | 238 | 0.2 | -0.0, 0.5 | 0.2 | -0.1, 0.5 |
|  | Paneth cells | 168 | -0.4 | -0.9, 0.1 | -0.1 | -0.5, 0.2 |
| Norovirus | Chronic inflammation (0-3) | 237 | 0.2 | 0.0, 0.3 | 0.1 | -0.0, 0.3 |
|  | Enterocyte injury (0-3) | 241 | 0.2 | 0.1, 0.3 | 0.2 | 0.1, 0.3 |
| Rotavirus | Chronic inflammation (0-3) | 238 | 0.2 | -0.0, 0.5 | 0.1 | -0.2, 0.3 |
|  | Goblet cell depletion (0-4) | 241 | -0.3 | -0.7, 0.0 | -0.3 | -0.6, 0.1 |
|  | Paneth cell depletion (0-3) | 168 | 0.4 | -0.1, 1.0 | 0.4 | -0.1,0.8 |
| *Cryptosporidium* | Goblet cell depletion (0-4) | 243 | -0.4 | -0.6, -0.1 | -0.2 | -0.5, 0.0 |

Associations at the p<0.1 level are presented for univariate analyses as this was an exploratory analysis. These were included in multivariable analyses adjusted for age, time difference between stool collection and biopsy, and study center; only associations at the p<0.05 level were considered statistically significant in the multivariable regressions. 95% CIs are presented for both univariate and multivariable analyses for comparison purposes. β coefficients can be interpreted as a higher (+ coefficient) or lower (- coefficient) histology parameter score when a pathogen was detected compared to not detected. For example, in the multivariable models, the TSP-5 score was 3.2 percentage points (the TSP-5 ranges from 0-100%) higher and the IELs score (which ranges 0-4) was 0.2 higher when *Campylobacter* was detected compared to when it was not, holding covariates constant.

Abbreviations: BEECH, Biomarkers of Environmental Enteropathy in Children; BEED, Bangladesh Environmental Enteric Dysfunction; CI, confidence interval; IELs, intraepithelial lymphocytes; SEEM, Study of Environmental Enteropathy and Malnutrition; TSP-5, total score percent-5; VA, villus architecture

**Supplemental Table 5. Regression results presented with standardized β coefficients, BEECH, BEED, and SEEM.**

|  |  | Univariate | | | Multivariable | |
| --- | --- | --- | --- | --- | --- | --- |
| Pathogens | Histologic Parameters | N | Standardized β coefficient | 95% CI | Standardized β coefficient | 95% CI |
| *Campylobacter* | TSP-5 | 202 | 0.2 | -0.0, 0.6 | 0.3 | 0.0, 0.6 |
|  | Chronic inflammation | 240 | 0.2 | -0.0, 0.5 | 0.2 | -0.0, 0.5 |
|  | IELs | 243 | 0.2 | -0.0, 0.5 | 0.2 | 0.0, 0.5 |
| *Clostridioides difficile* | TSP-5 | 202 | -0.5 | -1.0, -0.0 | -0.4 | -0.9, 0.0 |
|  | Enterocyte injury | 244 | -0.4 | -0.8, 0.0 | -0.4 | -0.8, 0.0 |
|  | VA | 178 | -0.6 | -1.1, -0.0 | -0.5 | -1.0, 0.1 |
| *Enterotoxigenic E. coli* (ETEC) | TSP-5 | 202 | 0.3 | 0.0, 0.6 | 0.3 | 0.0, 0.6 |
|  | Chronic inflammation | 240 | 0.3 | 0.1, 0.6 | 0.2 | -0.0, 0.5 |
|  | Intramucosal Brunner’s glands | 238 | -0.2 | -0.5, 0.0 | -0.2 | -0.4, 0.1 |
|  | Paneth cell depletion | 168 | 0.4 | 0.1, 0.7 | 0.2 | -0.0, 0.5 |
| *Salmonella* | Chronic inflammation | 240 | 0.5 | 0.1, 0.8 | 0.2 | -0.3, 0.7 |
|  | IELs | 243 | -0.8 | -1.1, -0.4 | -0.3 | -0.8, 0.2 |
|  | Goblet cell depletion | 243 | -0.3 | -0.6, 0.0 | -0.2 | -0.7, 0.2 |
|  | Paneth cell depletion | 168 | 0.4 | 0.0, 0.8 | 0.2 | -0.3, 0.6 |
| Shiga toxin producing *E. coli* | Goblet cell depletion | 243 | -0.6 | -1.1, -0.1 | -0.3 | -0.8, 0.1 |
| *Shigella* | TSP-5 | 202 | 0.3 | 0.0, 0.6 | 0.3 | -0.0, 0.6 |
|  | Chronic inflammation | 240 | 0.4 | 0.1, 0.6 | 0.2 | -0.0, 0.5 |
|  | Enterocyte injury | 244 | 0.3 | 0.1, 0.6 | 0.4 | 0.1, 0.7 |
|  | Paneth cell depletion | 168 | 0.4 | 0.1, 0.8 | 0.2 | -0.1, 0.5 |
| Adenovirus 40/41 | Chronic inflammation | 240 | 0.4 | -0.0, 0.7 | 0.3 | -0.1, 0.7 |
|  | Intramucosal Brunner’s glands | 238 | 0.3 | -0.1, 0.7 | 0.3 | -0.1, 0.7 |
|  | Paneth cells | 168 | -0.4 | -0.8, 0.0 | -0.1 | -0.5, 0.2 |
| Norovirus | Chronic inflammation | 237 | 0.3 | 0.1, 0.6 | 0.2 | -0.0, 0.5 |
|  | Enterocyte injury | 241 | 0.4 | 0.2, 0.7 | 0.4 | 0.2, 0.7 |
| Rotavirus | Chronic inflammation | 238 | 0.4 | -0.1, 0.8 | 0.1 | -0.3, 0.6 |
|  | Goblet cell depletion | 241 | -0.4 | -0.9 0.0 | -0.3 | -0.8, 0.1 |
|  | Paneth cell depletion | 168 | 0.4 | -0.1, 0.9 | 0.3 | -0.1, 0.8 |
| *Cryptosporidium* | Goblet cell depletion | 243 | -0.5 | -0.8, -0.2 | -0.3 | -0.6, 0.0 |

Associations at the p<0.1 level are presented for univariate analyses as this was an exploratory analysis. These were included in multivariable analyses adjusted for age, time difference between stool collection and biopsy, and study center; only associations at the p<0.05 level were considered statistically significant in the multivariable regressions. 95% CIs are presented for both univariate and multivariable analyses for comparison purposes. Standardized β coefficients can be interpreted as a higher (+ coefficient) or lower (- coefficient) standard deviation of the histology parameter score when a pathogen was detected compared to not detected. For example, in the multivariable models, the TSP-5 score was 0.3 standard deviations (SD) higher and the IEL score was 0.2 SD higher when *Campylobacter* was detected compared to when it was not, holding covariates constant.

Abbreviations: BEECH, Biomarkers of Environmental Enteropathy in Children; BEED, Bangladesh Environmental Enteric Dysfunction; CI, confidence interval; IELs, intraepithelial lymphocytes; SD, standard deviation; SEEM, Study of Environmental Enteropathy and Malnutrition; TSP-5, total score percent-5; VA, villus architecture

**Supplemental Table 6: Regression results, BEED and SEEM.**

|  |  |  | Univariate | | Multivariable | |
| --- | --- | --- | --- | --- | --- | --- |
| Pathogen | Histologic Parameters | N | β coefficient | 95% CI | β coefficient | 95% CI |
| *Aeromonas* | TSP-5 (0-100%) | 137 | 10.9 | 2.7, 19.0 | 8.1 | 0.5, 15.6 |
|  | Enterocyte injury (0-3)  IELs (0-4) | 177  176 | 0.3  0.5 | 0.1, 0.6  -0.0, 1.0 | 0.4  0.5 | 0.2, 0.6  0.0, 1.0 |
|  | Paneth cell depletion (0-3) | 112 | 0.9 | 0.2, 1.6 | 0.4 | -0.1, 0.9 |
| *Campylobacter*^1^ | IELs (0-4) | 176 | 0.3 | 0.0, 0.5 | 0.3 | 0.0, 0.5 |
| *Campylobacter*, pan | IELs (0-4) | 176 | 0.3 | 0.1, 0.6 | 0.3 | 0.0, 0.5 |
| *C.* *jejuni* or *coli* | IELs (0-4) | 175 | 0.3 | 0.0, 0.5 | 0.3 | 0.0, 0.5 |
| *Clostridioides difficile* | TSP-5 (0-100%) | 137 | -7.2 | -14.5, 0.0 | -5.9 | -12.5, 0.7 |
|  | Enterocyte injury (0-3) | 177 | -0.2 | -0.3, -0.0 | -0.2 | -0.3, -0.0 |
|  | VA (0-4) | 116 | -0.7 | -1.4, -0.0 | -0.6 | -1.3, 0.1 |
| Enteroaggregative *E. coli* (EAEC) | IELs (0-4) | 176 | 0.3 | -0.0, 0.5 | 0.2 | -0.1, 0.5 |
| EAEC, *aaiC* | Chronic inflammation (0-3) | 173 | 0.1 | -0.0, 0.3 | 0.2 | -0.0, 0.3 |
|  | IELs (0-4) | 175 | 0.2 | -0.0, 0.5 | 0.2 | -0.0, 0.5 |
| EAEC, *aatA* | Epithelial detachment (0-4) | 176 | 0.2 | -0.0, 0.4 | -0.1 | -0.3, 0.1 |
| Typical Enteropathogenic *E. coli* (EPEC) | IELs (0-4) | 176 | 0.4 | 0.0, 0.7 | 0.3 | 0.0, 0.7 |
|  | Intramucosal Brunner’s glands (0-3) | 171 | -0.3 | -0.6, -0.0 | -0.3 | -0.6, -0.0 |
| Enterotoxigenic *E. coli* (ETEC) | TSP-5 (0-100%) | 137 | 4.5 | -0.2, 9.3 | 4.6 | -0.0, 8.6 |
|  | IELs (0-4) | 176 | 0.3 | 0.0, 0.5 | 0.3 | 0.1, 0.6 |
|  | Paneth cell depletion (0-3) | 112 | 0.4 | -0.0, 0.8 | 0.3 | -0.1, 0.6 |
| Heat stable ETEC^2^ | IELs (0-4) | 176 | 0.3 | -0.0, 0.6 | 0.4 | 0.1, 0.8 |
| Heat stable ETEC, STh | IELs (0-4) | 175 | 0.4 | 0.0, 0.7 | 0.4 | 0.1, 0.7 |
| Atypical EPEC | IELs (0-4) | 176 | -0.2 | -0.5, 0.0 | -0.2 | -0.5, 0.1 |
| Shiga toxin producing *E. coli* | Goblet cell depletion (0-4) | 177 | -0.6 | -1.1, -0.1 | -0.3 | -0.8, 0.2 |
|  | Paneth cell depletion (0-3) | 112 | -0.8 | -1.7, 0.1 | -0.1 | -0.8, 0.6 |
| Shiga toxin producing *E. coli stx1* | Goblet cell depletion (0-4) | 176 | -0.7 | -1.2, -0.1 | -0.4 | -0.9, 0.1 |
| *Helicobacter pylori* | Epithelial detachment (0-4) | 177 | -0.5 | -1.0, 0.1 | -0.5 | -1.0, 0.1 |
| *Plesiomonas shigelloides* | TSP-5 (0-100%) | 137 | 10.9 | -0.5, 22.3 | 6.8 | -3.9, 17.6 |
|  | Epithelial detachment (0-4) | 177 | 0.6 | 0.1, 1.0 | 0.6 | 0.1, 1.0 |
|  | Goblet cell depletion (0-4 | 177 | 0.5 | -0.1, 1.1 | 0.3 | -0.3, 0.8 |
|  | Paneth cell depletion (0-3) | 112 | 1.0 | -0.1, 2.1 | 0.6 | -0.3, 1.4 |
|  |  |  |  |  |  |  |
| *Shigella* | TSP-5 (0-100%) | 137 | 6.4 | 0.5, 12.2 | 3.3 | -3.3, 9.8 |
|  | Paneth cell depletion (0-3) | 112 | 0.4 | -0.1, 0.9 | 0.3 | -0.2, 0.8 |
| Adenovirus 40/41 | Paneth cell depletion (0-3) | 112 | -0.8 | -1.3, -0.2 | -0.2 | -0.7, 0.2 |
| Astrovirus | IELs (0-4) | 174 | 0.5 | -0.1, 1.0 | 0.4 | -0.1, 0.9 |
| Norovirus | Enterocyte injury (0-3) | 174 | 0.1 | 0.0, 0.3 | 0.1 | 0.0, 03 |
|  | IELs (0-4) | 173 | 0.4 | 0.1, 0.7 | 0.4 | 0.1, 0.7 |
| Norovirus G1 | Goblet cell depletion (0-4) | 175 | -0.5 | -1, -0.1 | -0.4 | -0.8, 0.1 |
| Norovirus G2 | Chronic inflammation (0-3) | 172 | 0.2 | 0.0, 0.4 | 0.2 | 0.0, 0.4 |
|  | Enterocyte injury (0-3) | 175 | 0.1 | 0.0, 0.3 | 0.1 | 0.0, 0.3 |
|  | IELs (0-4) | 174 | 0.5 | 0.2, 0.8 | 0.4 | 0.2, 0.7 |
| *Cryptosporidium* | Goblet cell depletion (0-4) | 177 | -0.4 | -0.8, -0.1 | -0.3 | -0.6, 0.0 |
|  | Paneth cell depletion (0-3) | 112 | -0.5 | -1.0, -0.0 | -0.2 | -0.5, 0.2 |
| *E. bienuesi* | TSP-5 (0-100%) | 137 | 7.4 | -0.0, 14.9 | 6.3 | -0.4, 13.1 |
|  | Enterocyte injury (0-3) | 177 | 0.2 | -0.0, 0.4 | 0.2 | -0.0, 0.4 |
|  | IELs (0-4) | 176 | 0.5 | 0.0, 0.9 | 0.5 | 0.0, 0.9 |
| Pathogen burden | Chronic inflammation (0-3) | 174 | 0.0 | -0.0, 0.1 | 0.0 | 0.0, 0.1 |
|  | Enterocyte injury (0-3) | 177 | 0.0 | 0.0, 0.1 | 0.0 | 0.0, 0.1 |
|  | IELs (0-4) | 176 | 0.2 | 0.1, 0.2 | 0.2 | 0.1, 0.2 |
| Virulence locus burden | Chronic inflammation (0-3) | 174 | 0.0 | -0.0, 0.1 | 0.0 | 0.0, 0.1 |
|  | Enterocyte injury (0-3) | 177 | 0.0 | 0.0, 0.0 | 0.0 | -0.0, 0.0 |
|  | IELs (0-4) | 176 | 0.1 | 0.1, 0.2 | 0.1 | 0.1, 0.2 |

Associations at the p<0.1 level are presented for univariate analyses as this was an exploratory analysis. These were included in multivariable analyses adjusted for age, time difference between stool collection and biopsy, and study center; only associations at the p<0.05 level were considered statistically significant in the multivariable regressions. 95% CIs are presented for both univariate and multivariable analyses for comparison purposes.

β coefficients for individual pathogens can be interpreted as a higher (+ coefficient) or lower (- coefficient) histology parameter score when a pathogen was detected compared to not detected. For example, the TSP-5 score was 8.1 (percentage) points (the TSP-5 ranges from 0-100%) higher and the enterocyte injury score (which ranges 0-3) was 0.4 higher when *Aeromonas* was detected compared to when it was not, holding covariates constant. β coefficients for burden variables represents mean higher/lower histology parameter scores with each one unit higher in the pathogen burden variable. For example, enterocyte injury score was 0.2 higher with every additional pathogen detected, holding covariates constant.

^1^Defined as targets *cpn60* (pan *Campylobacter*) or *cadF* (*C. coli* or *C. jejuni*) positive

^2^Defined as heat stable targets STp or STh positive

Abbreviations: BEED, Bangladesh Environmental Enteric Dysfunction; CI, confidence interval; IELs, intraepithelial lymphocytes; SEEM, Study of Environmental Enteropathy and Malnutrition; TSP-5, total score percent-5; VA, villus architecture

**Supplemental Table 7: Regression results presented with standardized β coefficients, BEED and SEEM.**

|  |  | Univariate | | | Multivariable | |
| --- | --- | --- | --- | --- | --- | --- |
| Pathogen | Histologic Parameters | N | Standardized β coefficient | 95% CI | Standardized β coefficient | 95% CI |
| *Aeromonas* | TSP-5 | 137 | 0.8 | 0.2, 1.4 | 0.6 | 0.0, 1.1 |
|  | Enterocyte injury | 177 | 0.9 | 0.4, 1.5 | 1.0 | 0.4, 1.6 |
|  | IELs | 176 | 0.6 | -0.0, 1.1 | 0.6 | 0.0, 1.2 |
|  | Paneth cell depletion | 112 | 0.8 | 0.2, 1.5 | 0.4 | -0.1, 0.8 |
| *Campylobacter*^1^ | IELs | 176 | 0.3 | 0.0, 0.6 | 0.3 | 0.0, 0.6 |
| *Campylobacter*, pan | IELs | 176 | 0.4 | 0.1, 0.7 | 0.4 | 0.1, 0.7 |
| *C.* *jejuni* or *coli* | IELs | 176 | 0.3 | 0.0, 0.6 | 0.3 | 0.0, 0.6 |
| *Clostridioides difficile* | TSP-5 | 137 | -0.5 | -1.0, 0.0 | -0.4 | -0.9, 0.1 |
|  | Enterocyte injury | 177 | -0.5 | -0.9, -0.0 | -0.5 | -0.9, -0.0 |
|  | VA | 116 | -0.6 | -1.2, -0.0 | -0.3 | -0.1, 0.7 |
| Enteroaggregative *E. coli* (EAEC) | IELs | 176 | 0.3 | -0.0, 0.6 | 0.3 | -0.1, 0.6 |
| EAEC, *aaiC* | Chronic inflammation | 173 | 0.3 | -0.1, 0.6 | 0.3 | -0.0, 0.6 |
|  | IELs | 175 | 0.3 | -0.0, 0.6 | 0.3 | -0.0, 0.6 |
| EAEC, *aatA* | Epithelial detachment | 176 | 0.3 | -0.0, 0.6 | 0.3 | -0.0, 0.6 |
| Typical Enteropathogenic *E. coli* (EPEC) | IELs | 176 | 0.4 | 0.0, 0.8 | 0.4 | 0.0, 0.8 |
|  | Intramucosal Brunner’s glands | 171 | -0.4 | -0.8, -0.0 | -0.4 | -0.8, -0.0 |
| Enterotoxigenic *E. coli* (ETEC) | TSP-5 | 137 | 0.3 | -0.0, 0.7 | 0.3 | -0.0, 0.7 |
|  | IELs | 176 | 0.3 | 0.0, 0.6 | 0.4 | 0.1, 0.7 |
|  | Paneth cell depletion | 112 | 0.3 | -0.0, 0.7 | 0.2 | -0.1, 0.5 |
| Heat stable  ETEC^2^ | IELs | 176 | 0.3 | -0.0, 0.7 | 0.4 | 0.0, 0.7 |
| Heat stable ETEC, STh | IELs | 175 | 0.5 | 0.1, 0.9 | 0.5 | 0.1, 0.9 |
| Atypical EPEC | IELs | 176 | -0.3 | -0.6, 0.0 | -0.3 | -0.6, 0.1 |
| Shiga toxin producing *E. coli* | Goblet cell depletion | 177 | -0.8 | -1.4, -0.1 | -0.4 | -1.0, 0.2 |
|  | Paneth cell depletion | 112 | -0.8 | -1.6, 0.1 | -0.1 | -0.8, 0.5 |
| Shiga toxin producing *E. coli stx1* | Goblet cell depletion | 176 | -0.9 | -1.5, -0.2 | -0.5 | -1.1, 0.1 |
| *Helicobacter pylori* | Epithelial detachment | 177 | -0.7 | -1.5, 0.1 | -0.7 | -1.5, 0.1 |
| *Plesiomonas shigelloides* | TSP-5 | 137 | 0.8 | -0.0, 1.6 | 0.5 | -0.3, 1.2 |
|  | Epithelial detachment | 177 | 0.8 | 0.1, 1.6 | 0.8 | 0.1, 1.6 |
|  | Goblet cell depletion | 177 | 0.6 | -0.1, 1.3 | 0.4 | -0.3, 1.0 |
|  | Paneth cell depletion | 112 | 0.9 | -0.1, 1.9 | 0.6 | -0.2, 1.3 |
| *Shigella* | TSP-5 | 137 | 0.5 | 0.0, 0.9 | 0.3 | -0.1, 0.7 |
|  | Paneth cell depletion | 112 | 0.4 | -0.1, 0.9 | 0.2 | -0.2, 0.5 |
| Adenovirus 40/41 | Paneth cell depletion | 112 | -0.7 | -1.2, -0.2 | -0.2 | -0.6, 0.2 |
| Astrovirus | IELs | 174 | 0.5 | -0.1, 1.2 | 0.5 | -0.2, 1.1 |
| Norovirus | Enterocyte injury | 174 | 0.4 | 0.0, 0.7 | 0.4 | 0.0, 0.7 |
|  | IELs | 173 | 0.5 | 0.1, 0.8 | 0.5 | 0.1, 0.8 |
| Norovirus G1 | Goblet cell depletion | 175 | -0.6 | -1.2, -0.0 | -0.4 | -1.0, 0.1 |
| Norovirus G2 | Chronic inflammation | 172 | 0.4 | 0.1, 0.8 | 0.4 | 0.1, 0.8 |
|  | Enterocyte injury | 175 | 0.4 | 0.0, 0.7 | 0.4 | 0.0, 0.8 |
|  | IELs | 174 | 0.5 | 0.2, 0.9 | 0.5 | 0.2, 0.9 |
| *Cryptosporidium* | Goblet cell depletion | 177 | -0.5 | -0.9, -0.1 | -0.3 | -0.7, 0.1 |
|  | Paneth cell depletion | 112 | -0.5 | -0.9, -0.0 | -0.1 | -0.5, 0.2 |
| *E. bienuesi* | TSP-5 | 137 | 0.5 | -0.0, 1.1 | 0.5 | -0.0, 0.9 |
|  | Enterocyte injury | 177 | 0.4 | -0.1, 0.9 | 0.5 | -0.0, 1.0 |
|  | IELs | 176 | 0.5 | 0.0, 1.0 | 0.6 | 0.1, 1.1 |
| Pathogen burden | Chronic inflammation | 174 | 0.1 | -0.0, 0.2 | 0.1 | 0.0, 0.2 |
|  | Enterocyte injury | 177 | 0.1 | 0.0, 0.2 | 0.1 | 0.0, 0.2 |
|  | IELs | 176 | 0.2 | 0.1, 0.3 | 0.2 | 0.1, 0.3 |
| Virulence locus burden | Chronic inflammation | 174 | 0.1 | -0.0, 0.1 | 0.1 | 0.0, 0.1 |
|  | Enterocyte injury | 177 | 0.1 | 0.0, 0.1 | 0.1 | -0.0, 0.1 |
|  | IELs | 176 | 0.1 | 0.1, 0.2 | 0.2 | 0.1, 0.2 |

Associations at the p<0.1 level are presented for univariate analyses as this was an exploratory analysis. These were included in multivariable analyses adjusted for age, time difference between stool collection and biopsy, and study center; only associations at the p<0.05 level were considered statistically significant in the multivariable regressions. 95% CIs are presented for both univariate and multivariable analyses for comparison purposes.

Standardized β coefficients for individual pathogens can be interpreted as a higher (+ coefficient) or lower (- coefficient) standard deviation (SD) of the histology parameter score when a pathogen was detected compared to not detected. For example, in multivariable models the TSP-5 score was 0.6 standard deviations higher and the enterocyte injury score was 1.0 SD higher when *Aeromonas* was detected compared to when it was not, holding covariates constant. β coefficients for burden variables represent higher/lower histology parameter standard deviations with each one unit higher in the pathogen burden variable. For example, in multivariable models the enterocyte injury score was 0.2 SD higher with every additional pathogen detected, holding covariates constant.

^1^Defined as targets *cpn60* (pan *Campylobacter*) or *cadF* (*C. coli* or *C. jejuni*) positive

^2^Defined as heat stable targets STp or STh positive

Abbreviations: BEED, Bangladesh Environmental Enteric Dysfunction; CI, confidence interval; IELs, intraepithelial lymphocytes; SD, standard deviation; SEEM, Study of Environmental Enteropathy and Malnutrition; TSP-5, total score percent-5; VA, villus architecture
